# Supplementary material for: Long-Term Straw Returning Enhances Phosphorus Uptake by Zea mays L. through Mediating Microbial Biomass Phosphorus Turnover and Root Functional Traits
Source: Plants (Basel). 2024 Aug 27;13(17):2389. doi: 10.3390/plants13172389 (PMC11397705; doi:10.3390/plants13172389)
Supplement: Supplementary file 1 [file plants-13-02389-s001.zip › plants-3149512-supplementary.pdf]

# Supplementary information

**Table S1** The primers used for quantitative qPCR and corresponding amplification cycling conditions.

| Primer set | Target gene | Amplicon length (bp) | Amplificatio efficiencies | Amplification cycling conditions | References |
|---|---|---|---|---|---|
| ALPS-F730 (CAGTGGGACGACCACGAGGT)<br>ALPS-1101 (GAGGCCGATCGGCATGTCG) | *phoD* | 371 | 103% | 40 cycle (95 °C 30 s, 60 °C 5 s, 72°C 34 s) | Janssen, 2006;<br>Luo et al. (2019) |
| phoC-A-F1 (CGGCTCCTATCCGTCCGG)<br>phoC-A-R1 (CAACATCGCTTTGCCAGTG) | *phoC* | 155 | 99% | 40 cycle (95 °C 30 s, 58 °C 30 s, 72°C 20 s) | Gaiero et al. (2018)<br>Fraser et al. (2017) |

## References


Fraser, T. D., Lynch, D. H., Gaiero, J., Khosla, K., Dunfield, K. E. (2017). Quantification of bacterial non-specific acid (*phoC*) and alkaline (*phoD*) phosphatase genes in bulk and rhizosphere soil from organically managed soybean fields. Applied soil ecology, 111, 48-56.

Gaiero, J. R., Bent, E., Fraser, T. D., Condron, L. M., Dunfield, K. E. (2018). Validating novel oligonucleotide primers targeting three classes of bacterial non-specific acid phosphatase genes in grassland soils. Plant and Soil, 427, 39-51.

Janssen, P. H. (2006). Identifying the dominant soil bacterial taxa in libraries of *16S* rRNA and *16S* rRNA genes. App. Environ. Microb. 72, 1719-1728. doi: 10.1128/AEM.72.3.1719-1728.2006

Luo, G., Sun, B., Li, L., Li, M., Liu, M., and Zhu, Y., et al. (2019). Understanding how long-term organic amendments increase soil phosphatase activities: insight into *phoD*- and *phoC*-harboring functional microbial populations. Soil Biol. Biochem. 139, 107632. doi: 10.1016/j.soilbio.2019.107632

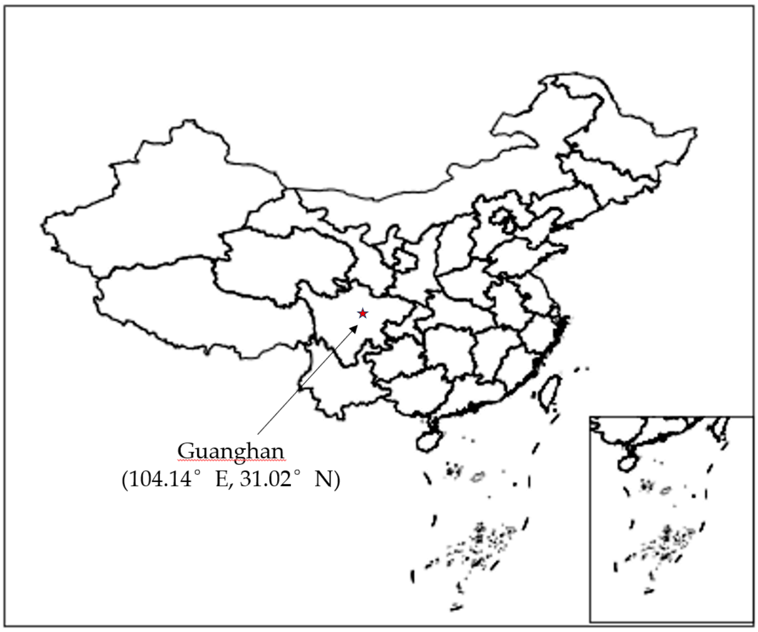


Fig. S1 Experimental site location

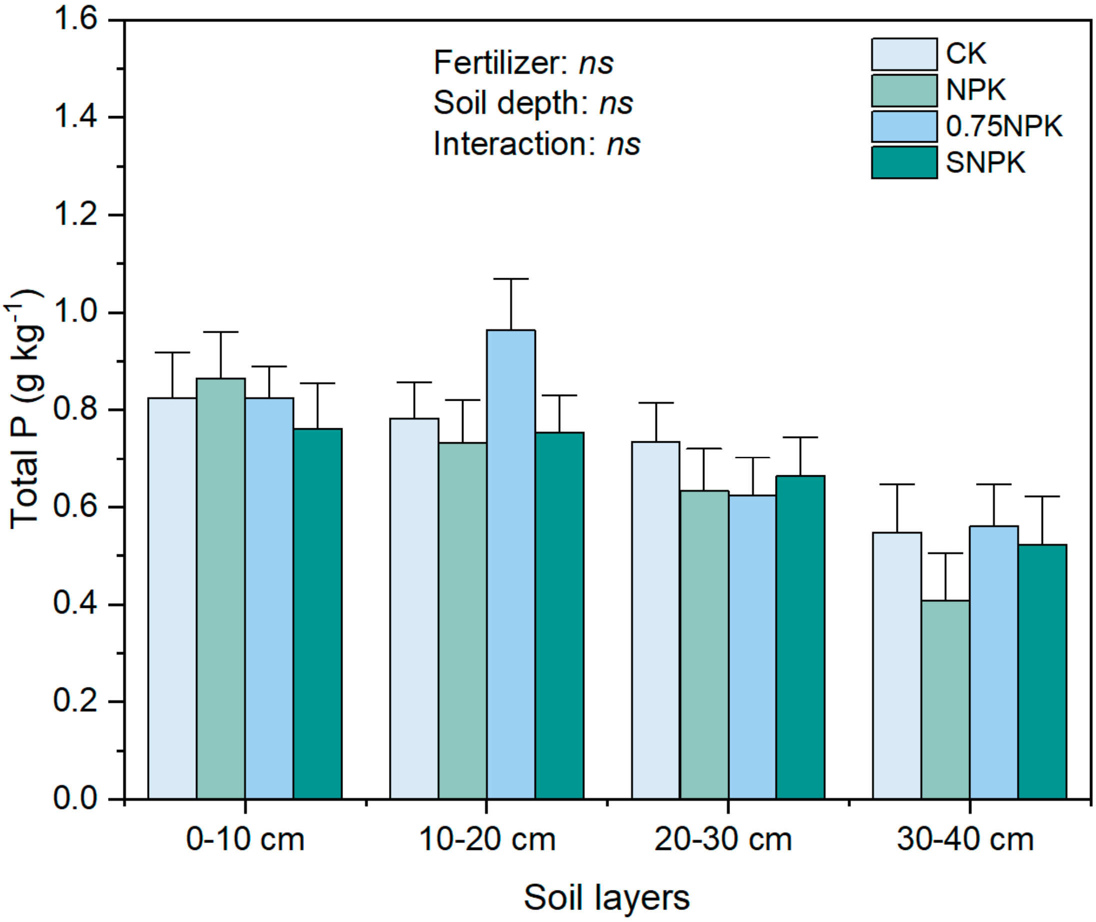


Fig. S2 Changes of total phosphorus under different fertilization treatments and soil depth. Two-way ANOVA was performed with fertilization treatment and soil depth as fixed effects. Different lower-case letters indicate significant differences ($p < 0.05$) among treatments at the same soil depth.
